# Conceptualizations of Knowledge in Structuring Approaches to Moral Development: A Process-Relational Approach

**DOI:** 10.3389/fpsyg.2021.756654

**Published:** 2021-12-16

**Authors:** Jeremy I. M. Carpendale, Vicki L. Parnell, Beau Wallbridge

**Affiliations:** Department of Psychology, Simon Fraser University, Burnaby, BC, Canada

**Keywords:** moral development, worldviews, knowledge, nativism, empiricism, constructivism, process-relational

## Abstract

Like other aspects of child development, views of the nature and development of morality depend on philosophical assumptions or worldviews presupposed by researchers. We analyze assumptions regarding knowledge linked to two contrasting worldviews: Cartesian-split-mechanistic and process-relational. We examine the implications of these worldviews for approaches to moral development, including relations between morality and social outcomes, and the concepts of information, meaning, interaction and computation. It is crucial to understand how researchers view these interrelated concepts in order to understand approaches to moral development. Within the Cartesian-split-mechanistic worldview, knowledge is viewed as representation and meaning is mechanistic and fixed. Both nativism and empiricism are based in this worldview, differing in whether the source of representations is assumed to be primarily internal or external. Morality is assumed to pre-exist, either in the genome or the culture. We discuss problems with these conceptions and endorse the process-relational paradigm, according to which knowledge is constructed through interaction, and morality begins in activity as a process of coordinating perspectives, rather than the application of fixed rules. The contrast is between beginning with the mind or beginning with social activity in explaining the mind.

## Introduction

Morality permeates our lives. It is embedded in our way of life, in ways that range from how we treat each other in everyday interaction, to broader social and political levels concerning injustice and inequality. The social fabric of our cultural worlds is woven around how we treat each other, and concern for others and their dignity is embedded in the pragmatic structures of human communication. Morality extends to larger scale social structures and decisions that affect others’ lives and involves concern for others’ welfare and well-being and obligations concerning what is right and wrong (Dahl and Killen, 2018). We face decisions that affect others at many levels, and we now have increasing awareness of how our actions affect others around the world through climate change. Thus, morality is a central aspect of being human and living with others.

The importance of morality in human life raises the question of how children become moral. Accounting for this aspect of child development depends on understanding the philosophical assumptions regarding knowledge and meaning which are the starting points for developmental research (Jopling, 1993). If we want to understand how children come to think about the world, including moral aspects of experience, we cannot ignore how they come to know the world, and this requires an epistemological analysis concerning views about the nature and development of knowledge (Chapman, 1999). We analyze conceptions of knowledge assumed by researchers that are linked to two worldviews: Cartesian-split-mechanistic and process-relational (Overton, 2015). We explore how the related concepts of information, meaning, and interaction are conceptualized differently from the perspectives of these two worldviews, and play a role in the ways that morality is understood.

Two contrasting approaches to morality that tend to be taken for granted are the nativist view that moral norms are primarily explained as pre-existing in humans’ biological nature, versus the empiricist view that moral norms pre-exist in the social world and are imposed on children from previous generations. These are generally the only options considered, so that if researchers argue against nativism it is assumed that they are arguing for empiricism. Most researchers, whether they emphasize nativism or empiricism, acknowledge that morality is some synthesis of biological and cultural (e.g., Bloom, 2010; Mikhail, 2020). Clearly it is necessary to include biological and evolutionary factors as well as social and cultural dimensions in understanding moral development, but it is possible to explicate the role of biology either from a gene centered approach, as in nativism, or an alternative, developmental systems approach (Griffiths and Tabery, 2013) that we outline, consistent with a process-relational perspective.

We argue that it is not just a matter of knowing where to draw the line in a “middle ground” between nativism and empiricism. This does not solve the problem because nature and nurture are still taken as pre-existing; information is assumed to pre-exist, either in the genes or in the environment. Both approaches are problematic, in that rather than explaining moral norms they explain them away by reducing them to either biological determinism or conformity to culturally imposed rules. In both views the passive individual is caused to act so there is no moral agent making choices to act morally, and thus neither approach cannot explain the development of a sense of moral obligation (Carpendale et al., 2010).

Morality is a complex aspect of human life that can be discussed in terms of cognition, emotions, and action. Various theorists focus on different aspects of this interrelated system and conceptualize the processes differently. For instance, in reacting to Kohlberg’s emphasis on reasoning others have more recently swung to focus on emotions. However, their role in morality is conceptualized in radically different ways. One claim is that moral decisions are due to evolved emotional responses (Haidt, 2001). We recognize the important role of emotions, but we consider their role in structuring the relationships in which morality develops. From the developmental systems perspective we propose, although these aspects of morality can be abstracted, they are interrelated and bidirectionally interwoven over development. Morality concerns the coordination of actions with others, and cognition, emotions, and action are aspects of the interaction with others in which children develop morality.

From our perspective on understanding the links between morality and social consequences, morality emerges within lives lived with others, and thus, within social consequences. From the action-based perspective we endorse, morality emerges from social and emotional interaction, so social consequences underlie the development of morality. Once morality begins to emerge as a way of understanding and thinking about interaction with others, it then plays a role in individuals’ choices about actions. Skills in understanding and making moral choices can influence future moral action in a complex bidirectional manner that plays out over developmental time.

In contrast to the approaches we criticize, we suggest that explaining the development of moral thinking and action should begin with interpersonal relations from the perspective of a process-relational worldview. We trace the implications for minds and morality of the view that “We are what we are through our relations with others” (Mead, 1934, p. 379). We propose that moral norms emerge in intersubjective experience through cooperative interaction among equals. We outline a process-relational approach to explaining the emergence of moral norms, beginning in infancy within the human social and emotional developmental system and extending through childhood. Interpersonal agreement is made possible in relationships based on mutual affection and mutual respect. Within such cooperative relationships among equals, practical morality emerges before children are able to articulate and then think about moral conflicts. In this way morality has its developmental roots in interactivity, and as children master a language they can then use it to reflect on moral issues and make decisions. Thus, morality is understood as beginning in social and emotional activity before children are able to think about and reflect on moral issues. What emerges is not a set of moral rules but rather a method or process for reaching moral decisions (Piaget, 1932/1965; Mead, 1934; Carpendale et al., 2013)^1^.

We first outline and critique current nativist explanations of moral development, focusing primarily on Moral Foundations Theory (Graham et al., 2013). We then explore how the nativist-empiricist debate is embedded in the Cartesian-split-mechanistic worldview, and instead we suggest a developmental systems perspective. Then we consider how conceptions of meaning and knowledge are influenced by worldviews. Finally, as an alternative, we outline an approach to moral development from the perspective of the process-relational worldview^2^.

## Biology and Environment in Conceptualizations of Interaction and Information

There are many recent claims that some aspects of morality are innate (e.g., Hauser, 2006a,b; Hamlin et al., 2007; Mikhail, 2007, 2020; Bloom, 2010, 2012; Hamlin, 2013; Margolis and Laurence, 2013; Warneken, 2016). Bloom (2010, p. 46), for example, claimed that humans, “have a rudimentary moral sense from the very start of life.… Some sense of good and evil seems to be bred in the bone.” There is ongoing debate regarding this claim (e.g., Prinz, 2009; Sterelny, 2010), and elsewhere we have criticized nativist approaches making claims that infants are born with innate principles of fairness (Carpendale et al., 2021), an innate moral core (Carpendale et al., 2013), and innate altruism (Carpendale et al., 2015; Carpendale and Lewis, 2021). Here we focus our critical attention primarily on one highly cited approach and we check the foundations of Moral Foundation Theory (Graham et al., 2013).

According to Moral Foundations Theory, “genes (collectively) write the first draft into neural tissue, beginning *in utero* but continuing throughout childhood. Experience (cultural learning) revises the draft during childhood, and even (to a lesser extent) during adulthood”(Graham et al., 2013, p. 61). In stating their claim that morality is innate, Graham et al. (2013, p. 100) assert that, “the discussion should focus on how exactly moral knowledge is innate, not whether it is.” However, despite this strong claim, they and others do not discuss “how exactly moral knowledge is innate” (Dahl et al., 2021). In phrasing the problem in terms of *how* moral knowledge is innate, it seems that what is being proposed is an account of how knowledge could be innate, referring to the biological processes involved in getting from genes to justice and from neurons to norms. We suggest that an attempt to explicate *how* this is claimed to occur would make it clear that a direct route is in fact not consistent with current biology.

With its reference to the role of genes, nativism could mistakenly be taken for a biological approach, and to criticize nativism is thus to criticize the role of biological factors and instead argue for social factors. That is, it could be assumed that the only alternative to nativism’s claim that knowledge pre-exists in biology is empiricism and social factors. But this buys into the very dichotomy we reject. In discussing flaws in nativism we are not arguing for empiricism. In fact, nativism and empiricism share many of the same problematic foundations of knowledge as pre-existing, either in the individual or in the society.

In fact, nativism does not amount to taking biology seriously, and does not actually rest on biological knowledge. Although Graham et al. (2013) claim that genes write into neural tissue, they fail to provide a reference to the biological literature regarding this claimed process^3^. Nativists seem to have a tendency to neglect citing biological research; this is consistent with the tradition following from Chomsky (2007), who was so compelled by his logical arguments that evidence from psychology or biology was not considered relevant. In fact, a number of biologists and geneticists have attempted to provide psychologists with some rudimentary idea of how genes work in the hope that they might improve understanding of the role of genes and environment in development (e.g., Fisher, 2006; Meaney, 2010), as well as how neural pathways are formed through experience (e.g., Mareschal et al., 2007; Stiles, 2009; Stiles et al., 2015). Genes do not write into neural tissue in the sense of forming neural connectivity. There is a long “tortuous” process through which genes are involved in a system resulting in human development (e.g., Fisher, 2006).

Genes are fairly inert molecules involved in the production of chains of amino acids as a step in the production of proteins, but the chains still need to be folded up within the cell. Although genes are crucial in development, it is problematic to assume that information exists at the level of genes or a “genetic program” because how genes function depends on many other factors in the environment, beginning at the level of the cell and extending to social interaction. The effect of a gene can vary as widely as promoting cell life or leading to cell death, depending on what co-factors exist in the cytoplasm (Meaney, 2010). Thus, the foundations of MFT appear to be based on implausible biological assumptions. Furthermore, the incredible complexity of neural interconnectivity that makes human lives possible is gradually shaped through experience (e.g., Stiles, 2009; Stiles et al., 2015).

Although clearly it is necessary to include biological and evolutionary factors as well as social and cultural factors in understanding moral development, the process-relational approach we propose is fundamentally different from a mixture of nativism and empiricism. The approach we endorse does of course consider the human developmental system consisting of evolved biological characteristics of infants and parents, set within cultural contexts. However, there are two fundamentally different ways in which this interaction can be conceptualized: either through a gene-centered approach according to which these are considered separate, pre-existing factors that then interact, or from a developmental systems perspective that does without the dichotomy (e.g., Gottlieb, 2007; Griffiths and Tabery, 2013). We have discussed flaws with the gene-centered approach that assumes genes carry information in a genetic program; instead, we suggest that genes are one crucial factor in a system of bidirectionally interacting factors. Thus, in contrast to nativism, process-relational approaches take biology seriously through its role in human developmental systems. Rather than thinking of biology and environment as separable factors that interact, if we look closely at development, we see a system from which these interwoven factors are abstracted. Biology cannot be separated from environments because organisms create and elicit their environments through their characteristics, and, in turn, biology is structured by experience. This process begins with infants’ action tendencies and sensory abilities in early development, which elicit the environment in which they develop in a bi-directional manner.

Regularity in developmental outcomes could be assumed to be evidence for nativists’ claims of knowledge pre-existing at the genetic level. However, such regularity does not mean that it must be due to something pre-existing, such as genes. To illustrate this point, consider the example of mature forests in particular climate zones. The type of forest tends to be a consistent and regular outcome over many decades. This is the result of the interaction of multiple factors, including the characteristics of the species involved such as their tolerance for shade and so on. For example, a mature forest in the Pacific northwest tends to be Western Red Cedar and Douglas Fir, even though the information for that outcome does not pre-exist anywhere. Instead, it is the natural outcome of ecological succession within particular conditions (Griffiths and Stotz, 2000).

Rather than assuming pre-existing information encoded in genes, we argue that regularities in the development of moral knowledge can emerge through typical human developmental systems. We now turn from criticizing claims about getting from genes to the first draft of moral cognition, to analyzing the nature of this cognition.

## Conceptions of Knowledge and Meaning in Contrasting Worldviews

The moral nativists we are focusing on assume the computational/representational framework. According to this computational theory of mind, thinking—in this case, about morality—is based on computation involving mental representations that are linked to the world (e.g., Graham et al., 2013; Mikhail, 2020). Moral Foundations Theory

*“proposes that the human mind is organized in advance of experience so that it is prepared to learn values, norms, and behaviors related to a diverse set of recurrent adaptive social problems…. We think of this innate organization as being implemented by sets of related modules which work together to guide and constrain responses to each particular problem”* (Graham et al., 2013, p. 63).

For example, it is assumed that infants are born with innate principles such as a principle of fairness in the “first draft” of moral cognition (e.g., Bian et al., 2018; Buyukozer Dawkins et al., 2019). From this perspective, thinking is computation based on mental representations that are meaningful because they are linked to the world.

An issue with this view, however, is the problem of how such representations are linked to the world in a way that makes them meaningful. As Wittgenstein (1953/2009) has argued, representations cannot carry their own meaning because any representation could be interpreted in multiple ways (e.g., Heil, 1981; Goldberg, 1991). Thus, the only way to bring meaning into the computational/representational framework is to implicitly assume a homunculus (Heil, 1981; Kenny, 1991). That is, what is required is something in the system like a small person that attributes meaning to the representation, just as a person must attribute meaning to the input and output of a computer. This, of course, is problematic because it just puts off rather than provides an explanation (for further criticism of the computational theory of mind see e.g., Heil, 1981; Carpendale et al., 2021; Carpendale and Lewis, 2021).

As Hobson (2002, p. 14) noted, “computers don’t understand anything, nor do they care.” To further spell out Hobson’s point, computers don’t understand *because* they don’t care. A computational system is not linked to the world in ways that can involve significance and meaning. Morality, however, is necessarily based on the emotional significance of social actions and outcomes for the people involved. It involves coordinating conflicting goals.

A mechanical process is not based on meaning, so it is not possible to get from computation to caring. To claim that a computer program can contain principles of fairness is like assuming that an automatic door opener is polite. The door works through a passive mechanical process. If it fails to open it is not rude, just broken. In contrast, persons open doors for others because they are recognized as persons with goals and all that entails. Similarly, although it is possible to design a mechanism to divide resources equally, this does not mean that the machine is moral and is applying a principle of fairness. If it fails to operate properly it is not immoral, just defective. If morality is reduced to the mechanical computation that genes write into neural tissue, then it is causal not normative, and within such an approach the normative dimension of morality is not explained; rather, it is explained away. Additionally, this sort of an approach risks self-contradiction if theorists claim to be involved in the normative enterprise of science yet there is no place for reasons in their theory (Habermas, 1983/1990; Carpendale et al., 2010). This would be like “sawing off the branch on which one sits” (Bennett and Hacker, 2003, pp. 376–377). We have analyzed the influence of how information is conceptualized in the context of genes encoding information, and how meaning is conceptualized as fixed in the context of the computational view of the mind. Both conceptualizations of information and meaning in this Cartesian-split-mechanistic worldview are derived from the theory of knowledge as representation. Both nativism and empiricism, although apparently different approaches, actually begin from the same Cartesian-split-mechanistic worldview (Overton, 2015), and the theory of knowledge as pre-existing representation that is located either primarily in the child and explained through biology or in the social world and imposed on the child.

This conception of knowledge is what Piaget (1970) referred to as a copy theory and Dewey (1960) labeled the spectator notion of knowledge. Both descriptions bring out the point that knowledge is viewed as solely based on perception (Piaget, 1970/1972). According to this perspective, developing knowledge is viewed as forming a copy of the world. But because it is assumed that we have no direct access to the world, the only way to check our representation is by forming another copy (Wittgenstein, 1953/2009). Thus, we cannot tell if our representation of the world that is meant to reflect our knowledge of the world is accurate because we cannot directly compare it to the world. If we cannot become aware of errors then we cannot learn, and an approach that cannot account for learning is fundamentally flawed (Bickhard, 2009). Although such an approach is meant to explain knowledge, in fact, it already presupposes knowledge (e.g., Dewey, 1960; Piaget, 1970; Chapman, 1999; Carpendale and Lewis, 2004, 2006, 2021; Bibok et al., 2008; Bickhard, 2009).

We have argued that the worldview on which nativism and empiricism are based is problematic, and is based on a flawed view of knowledge as representation, which results in flawed conceptualizations of information and meaning. An alternative approach to knowledge, consistent with the process-relational worldview, is an emergent constructivism according to which knowledge develops through learning the interactive potential of the world. This world includes other people, which adds a normative dimension to this process. An infant is an agent with needs and emotions who learns how the world responds to her actions in positive or negative ways. Thus, meaning emerges along with anticipations as the child learns about the world and what she can do with it (Piaget, 1936/1952), and perception is seeing the world in terms of the potential for interaction (e.g., Chapman, 1999). An understanding of fairness is based on the meaning actions have in terms of the emotional consequences for the people involved, and others’ reactions must be valued. We now turn to explicating a view of moral development based on this perspective.

## A Process-Relational Approach to Moral Development

Moral norms concern what is right and wrong. They are not a part of the physical world, so how do they arise (Brandom, 1994)? We don’t observe an understanding of commitment, obligation, and right and wrong in other species. What, then, is the source of moral norms? Children grow up in cultures with moral norms that are imposed on them. They may come to understand and accept these moral rules, or perhaps challenge and possibly change such norms. To account for this and explain how it is that rather than passively accept rules children may challenge and attempt to change cultural norms, we argue that moral norms and right and wrong emerge at the level of action and interaction. Assumptions about the source of moral norms are linked to assumptions about their nature. That is, if they are causal through being determined by biology, or external and imposed from the outside, then it could be questioned whether they are actually morality because the individual is being compelled to act (Wright, 1982a,b). Thus, this way of thinking seems to define moral norms out of existence (Carpendale et al., 2010). Although neurons are necessary for morality, they do not cause norms and explain moral development. Just as we do not get an answer for why two plus two is four at the level of neural activity (Piaget, 1971, p. 49), morality does not arise at the level of the individual and biological activity.

The process-relational approach we propose begins from activity, and thus fits with Piaget’s (1932/1965) still overlooked view that children first work out a way of interacting with each other in their practical activity through coordinating their actions with others. Piaget began from practical interaction, within a particular form of relationship that is based on mutual affection and mutual respect. In these relationships, children enjoy the interaction and want to continue it and thus have to work out a way of getting along with each other and coordinating their sometimes conflicting goals. This is a form of interaction that is best suited to reaching mutual understanding because equals feel an obligation to listen to each other as well as explain their own position (Piaget, 1932/1965). There is already a form of morality in the “constitutive rules” that structure such relationships because the individuals involved treat each other as persons and listen to others as well as explain their own perspective. Thus, “morality is the logic of action” (Piaget, 1932/1965, p. 398). Within such interaction it is possible to formulate “constituted rules” concerning how to coordinate their action (Piaget, 1932/1965; Carpendale, 2009).

This outcome is a coordination of everyone’s interests, and it must be based on caring about each other as a foundation in structuring the interaction. Caring is not something that is added later or reached through reasoning. Instead, concern for others and not just taking everything for oneself is part of the foundation of the human developmental system. This involves treating others as persons—someone, not something (Spaemann, 2006). This interaction is based on care, affection, and enjoyment of interaction. Language is used in order to explain oneself and listen to others in coordinating conflicting goals.

Once a form of morality has emerged at the practical, lived level, Piaget (1932/1965) then suggested that a gradual process of “conscious realization” occurs, through which children became able to verbally articulate the ways of interacting that are already present in their activity. Here language plays an important role, first, in the process of reaching mutual understanding within interaction among equals and achieving a solution to conflicts that is agreeable to all. Second, language again plays a role in the process of articulating and reflecting on that earlier achieved competence in the way children treat each other. From this perspective morality does not begin in the structure of the mind. Instead, it begins in social activity and emerges in the coordination of action through experiencing the consequences of one’s actions. The mind is structured through activity and cannot be structured prior to experience.

From this perspective, morality is a process. There is nothing “objective” about this in a foundational or fixed sense of certainty outside of human interaction. Morality is also not “subjective” in the sense of just being based on personal whims. Instead, it is intersubjective in the sense of arising through coordination with others. It is the logic of interaction that arises in the coordination of action with others (Piaget, 1932/1965). And in this sense, it is an aspect of communication and cooperation through being based on valuing all other viewpoints as equal. This begins by being rooted in relations based in mutual affection and caring for each other. Children first work out a practical morality as a way of coordinating their action with others because it is more enjoyable to play that way. Adults can play a role in facilitating relationships that are best suited to reaching understanding. But this is different from adults imposing rules that children don’t understand.

Similar ideas are present in the work of other theorists. The idea of coordinating conflicting perspectives was also present in Mead’s (1934, p. 389) argument for a moral process that involves considering all perspectives involved—“the method of morality.” Mead (1934, p. 379) derived the universality of moral judgment from our social nature, “from the fact that we take the attitude of the entire community, of all rational beings”… “that is, everyone who can rationally appreciate the situation agrees.” Moral norms should be grounded in good reasons that cannot be rationally rejected by anyone involved (Mead, 1934). Kohlberg had a similar perspective with his idea of moral development as ideal role taking, and his notion of “moral musical chairs” consisting of taking all the perspectives involved in a moral dilemma. But this was overshadowed by Kohlberg’s unfortunate adoption of a problematic view of Piaget’s stages (Carpendale, 2000). Habermas (1983/1990) argued that aspects of this process of considering all relevant points of view are embedded in the structure of conversation and argumentation, and thus engaging in these activities presupposes morality (see also Forst, 2005).

Piaget’s (1932/1965) account focuses on interaction among school aged children, but even to get to this point in development we suggest that it is already possible to see the beginning of morality in the preconditions for interaction and communication (Winch, 1972). Infants’ biological embodiment, such as being helpless at birth and thus requiring care, structures the social and emotional system in which they develop (Portmann, 1944/1990; Carpendale and Lewis, 2021). Here we focus on the moral aspects of the human developmental system. The roots of morality are already emerging in the way in which caregivers respond to their infants as persons. Even in the first few months of life mothers find it difficult to treat their babies as objects when researchers ask them to hold a “still face” rather than respond normally to their young infants. If infants are accustomed to the enjoyable interaction, they often try to elicit it by smiling if it is missing. Although caregivers’ difficulty in not responding to their babies is considered an obstacle in conducting research using the “still face” paradigm, from our perspective it is a finding revealing the way caregivers respond to their infants as persons (Mcquaid et al., 2009). This reciprocal responding to each other may be a source of the expectation for turn taking in interaction. Treating others as someone not something is already present in this early interaction. A foundational component for human declarative communication is the development of children’s enjoyment in participating in interaction as a goal in itself. This development can be charted from enjoying attention to self, then to what the self does, and to the activity of showing and giving objects, as well as also to interest and enjoyment in participating in adults’ activities (Bates et al., 1975; Rheingold, 1982; Reddy, 2003).

We begin from children’s activity within a developmental system, thus our approach could be mistaken for behaviorism. But our goal is to explain psychological development as arising from interaction and communication, rather than assume the mind as presupposed and therefore not explained (e.g., Mead, 1934). Behaviorism is restricted to passive association and cannot account for meaning. Thus, behaviorism is situated within the Cartesian-split-mechanistic worldview that we have criticized. Instead, from an action-based process approach, infants learn about the world through their experience within which they come to anticipate outcomes of their actions. They perceive the world in terms of potential for interaction (e.g., Chapman, 1999), and through this process the world becomes meaningful to them. Instead of a passive association, any association is due to the meaningful relationship the child forms. From an action-based perspective, the child is not thought of as learning a response to a stimulus. Instead, the child is “coming to organize his activity in a particular way (which he can extend to other contexts) and coordinating this activity with the corresponding acts of the mother” (Clark, 1978, p. 240). The child is not mechanistically and passively forming associations between meaningless unrelated stimuli. Instead, she is active with goals, needs and interests, as she learns about the potential for action on the physical world. Infants’ interaction with the world becomes more complex as they come to anticipate the outcomes of their actions. When infants begin employing an action as a means to attain a goal, we can say they are acting with intention, typically beginning at about 8 months of age (Piaget, 1936/1952). Toward the middle of their second year, toddlers begin to coordinate action schemes implicitly or mentally. This is the beginning of one form of mental activity in which they can mentally anticipate the outcomes of their actions (Piaget, 1936/1952).

Unintentional communication is present in interaction even in infants’ early months of life because, for example, the crying of a newborn infant has significance for caregivers. An important transition is to intentional communication beginning toward the end of the first year of life. This occurs as infants learn the significance or meaning their actions have for others. They come to anticipate social outcomes of stable social structures, or routines, such as requests, responding to questions, or sharing attention. These shared social routines form stable structures of interaction with common expectations in which words can be used (Carpendale and Lewis, 2021).

The links between communication and morality are complex and bidirectional over development. Communication in the human intentional sense and morality are both located within and emerge from forms of interaction in which individuals develop and experience themselves in relation to others (e.g., Carpendale, 2018). Communication from this perspective is not a matter of transmitting meaning attached to symbols, words, or representations, as it is in the Cartesian-split-mechanistic worldview. Rather, communication is situated in the coordination of action and so it entails a view of selves in relations to others, that is, morality. Communication and morality are two sides to a coin, two ways of talking about human relations.

Language provides a way of talking about and reflecting on as well as understanding human activity with mental state terms. Beginning in their third year, children start to learn words referring to mental states such as want, know, forget and so on. These words refer to human activity (Carpendale and Lewis, 2015). Through experiencing others’ reactions to themselves, children come to take themselves as an object. That is, to take others’ perspective on their self, and thus to become able to reflect on their self, and now to have a self rather than just be a self (Mead, 1934). Their psychological language can now be used to reflect on their own experience as well as help in understanding others’ experience. This is a developmental outcome of a gradual process that begins in activity and interaction with others (Wittgenstein, 1953/2009; Canfield, 2007; Racine and Carpendale, 2008). This approach to the development of children’s social understanding contrasts with the causal psychological view that mental states underlie and cause outer behavior, assumed in the Cartesian perspective underlying much of the work on children’s theories of mind (see Carpendale and Lewis, 2004, 2006, 2015, 2021; Racine and Carpendale, 2008). Instead, mental states are logically linked to such action and cannot be identified independently (Racine and Carpendale, 2008).

The link between morality and communication can be seen in the way that cooperation is a principle that underlies and structures typical conversation because it is possible to derive additional meaning from utterances in conversation based on the assumption that others are cooperating in conveying meaning (Grice, 1975). Furthermore, Grice viewed conversation as a special case of human cooperative interaction in general. Although it is possible to use language to deceive others, lying is only possible because truth telling is the norm (Holiday, 1988).

Concern for others and their dignity is embedded in the pragmatics of conversation and politeness (Brown and Levinson, 1987; Turnbull, 2003). The care and concern for others that structures our interaction is also a foundation for the development of moral obligation (Carpendale, 2018; Carpendale and Lewis, 2020). Language is based on moral aspects of interaction and enables further complexity in morality through understanding and coordinating with others’ perspectives.

Some of the processes we have presupposed in moral development are based on individuals giving reasons to others and listening to others’ reasons. But in the casual world of natural science how do we find room for reasons? Reasons have no place in the lives of other animals, even social species. Why is it that humans give reasons and expect reasons from others? From the perspective we take, reasoning emerges within social relations, within interpersonal obligations to others because they are given to others (Kitchener, 2004): “Man is a rational being because he is a social being” (Mead, 1934, p. 379). Giving reasons to others and valuing others’ reasons and responding develops within interpersonal relations of caring and obligation. In some families, young children are given reasons even before they can understand them, whereas in other families they may be told to do things without reasons. Children must also learn when reasons are expected from them, often to explain actions in the context of social expectations. This requires understanding our actions in relation to others and appreciating the effect of our actions on others, which involves being able to take others’ perspectives on the self. In Mead’s (1934, p. 138) words, “The importance of what we term ‘communication’ lies in the fact that it provides a form of behavior in which the organism or the individual may become an object to himself.” This allows individuals to see their actions in relations to others.

This link between communication and morality is further explicated by Spaemann (2006, pp. 14–15):

*“To speak of oneself in the third person is to step out of the central position that all living things in nature occupy in relation to their environments, and to see oneself with other people’s eyes as something ‘out there’. For this one must adopt a point of view from outside of one’s own organic center. Morality is possible only with this capacity for self-objectification and self-relativization; only on these terms, too, is speech possible. Speech differs from the cries of living things in nature, in that it anticipates the standpoint of the one who is to hear what is spoken. When someone says, ‘I am in pain’, that statement is not merely a cry by other means. The immediate expression of pain must be suppressed, in order to form a communication about the pain as an event in the world and to make that communication intelligible to another.”*

This ability to take oneself as an object through appreciating others’ attitudes toward the self makes it possible to consider one’s own perspective and action in relation to others, and this is required in order to coordinate perspectives and arrive at moral solutions. This social process of seeing oneself in others’ eyes may also be important in coming to value and integrate the moral dimensions of oneself, that is, to develop one’s moral self (Krettenauer, 2013). The approach to this problem that is consistent with the process-relational framework we endorse is to conceptualize development in this area as an inter-personal social process that is based in lived interaction and crosses multiple domains of development (Krettenauer, 2013). The relationships of mutual respect in which practical morality emerges are also linked to a sense of self value and confidence, as well as moral and intellectual development (Wright, 1982b).

We have grappled with the task of explaining the development of moral norms. But, given the extent of injustice and oppression in the world, it could be argued that the development of morality does not always, or even typically, occur, so why does it go wrong? From our perspective, development occurs within a system and thus many factors can vary resulting in different outcomes. But the first step is to explain how morality is even possible. Furthermore, we have to explain how it is that we can recognize injustice when it occurs. In addition, one’s conception of how things can go wrong depends on one’s view of how they can go right. Thus, the first step is to explain how morality is possible.

Of course, not all relationships children experience are cooperative. Children’s early relationships may necessarily tend to be more constraining, and any relationship is some mixture of constraint based on one-sided respect and inequality, and cooperation based on mutual respect and equality (Piaget, 1932/1965). Children do sometimes constrain and bully others, and, on the other hand, parents can be cooperative to varying degrees. The process we have described can run off the rails in various ways, and through this experience children learn about the social consequences of their actions (e.g., Dahl et al., 2011). There are factors in human interaction such as greed leading to oppression and inequality, and language can be used to deceive and oppress others. But there is also constant struggle against such forces as individuals recognize oppression, inequality, and lack of fairness. We are inclined to be convinced by Piaget’s (1932/1965) hopeful stance that in spite of setbacks there is still the potential for gradual progressivity in societal change toward a more just world (Chapman, 1988). Based on Piaget’s position, we argue that moral progress on a societal level is made possible because, as part of the foundation of moral development, caring for others means learning to care for and respect others’ perspectives and to treat these perspectives as equivalent to our own. These are the constitutive rules that underlie engaging in a moral process in which norms can be constructed and changed based on negotiation and consensus within relationships of mutual affection and mutual respect. This is a social process which begins with caring for others in close relationships and can then be extended to engaging with and respecting other perspectives on a societal level. Such a process, originally rooted in close interpersonal relationships, provides one means for moral progress as more perspectives become coordinated at a societal level (Mead, 1934; Carpendale and Lewis, 2020, 2021). This progress could extend beyond the initial circle of close relationships to include more perspectives within one’s culture and beyond, and could also be extended to other species and the broader biosphere^4^.

## Discussion

Humans inhabit webs of moral obligations and commitments. We live with a sense of right and wrong, an understanding of what ought to be done in a world of norms, a space of justification. We have grappled with the issue of explaining the source of such moral norms, and accounting for how the feeling of obligation emerges in children’s lives as different from conformity to social conventions. Two common approaches are that moral norms are imposed on children by previous generations or that they are innate. We have argued that by themselves these explanations are incomplete and are attempts to explain away rather than explain moral norms. Nativists reduce morality to being caused to act due to biology and empiricists reduce morality to conformity to socially imposed rules, so either, or a combination of the two, rule out the person as actually making a choice. From our perspective, both are no longer talking about morality, but are instead referring either to something within the child compelling her to act or to conformity to social rules. Although children do grow up within cultures with moral norms imposed on them, this does not explain the source of such norms and children must still come to understand and perhaps challenge and change such norms. A middle ground as a mixture of the two still does not deal with morality.

The assumption that biological factors play a role in morality must be further spelled out (Dahl et al., 2021) and this can be done either from a gene-centered perspective or a developmental systems approach. From the perspective we propose, morality and the idea of justice emerges and does not pre-exist in either societal beliefs nor in the biology of the individual. It develops reliably given certain conditions, just as a whirlpool is a structure that emerges in the flow of liquids given certain conditions, although it does not pre-exist anywhere. The individual’s biological heritage results in the conditions in which the idea of justice can emerge. From this perspective, it is recognized that knowledge cannot be innate in the sense that it is directly the result of genes, but rather that there is a much more complex developmental system in which ideas about morality can develop (Piaget, 1932/1965; Carpendale, 2009; Carpendale et al., 2013). Thus, a third option in understanding the development of moral norms is the developmental systems approach within a process-relational perspective according to which biological and social factors do not simply pre-exist separately but are instead abstracted from social and emotional developmental systems in which they are intertwined and mutually create each other.

The way that the link between morality and social outcomes is conceptualized depends on the worldview researchers adopt. From the perspective we take, the goal is to trace a natural history of the development of moral norms through the increasingly complex forms of coordination emerging in dyads as children construct social and moral skills through their interaction with others. Morality concerns the coordination of action with others, and it emerges within the social consequences of children’s actions at the level of intersubjective engagement with others who we respect and care for. Within cooperative relationships among equals children work out what is fair at a practical lived level. Norms are first implicit in interaction and ways of treating others, and children gradually come to consciously realize the principles that underlie their practical interaction. This process of interpersonal coordination continues in more complex ways with language as children became aware of the implicit norms that structured their interaction, and with the development of reasoning and justifications. From a process-relational perspective, communication and morality are interwoven. Morality emerges as an aspect of living with others. It is not that care and morality had to evolve as something separate, but rather caring about each other is what makes us human. It structures the human developmental system, the human social emotional cradle in which children develop (e.g., Carpendale and Lewis, 2021). Morality and communication emerge out of human relations. From an action-based perspective we begin with social activity, and the social consequences of children’s action form the experience through which children develop morality. In a bi-directional manner, the understanding children develop in this process can then influence their subsequent thinking and action.

## Author Contributions

JC wrote the first draft. VP and BW revised and added to that draft. All authors contributed to the article and approved the submitted version.

## Conflict of Interest

The authors declare that the research was conducted in the absence of any commercial or financial relationships that could be construed as a potential conflict of interest.

## Publisher’s Note

All claims expressed in this article are solely those of the authors and do not necessarily represent those of their affiliated organizations, or those of the publisher, the editors and the reviewers. Any product that may be evaluated in this article, or claim that may be made by its manufacturer, is not guaranteed or endorsed by the publisher.

## Footnotes

1 Describing and explaining the emergence of more complex forms of organization is central to developmental psychology. However, the possibility of emergence is highly controversial in philosophy (O’Connor, 2020). One barrier to the possibility of emergence is the assumption that all definition permits back-translation. Bickhard (2009) claims, however, that this is false because implicit definition does not. A second barrier to the possibility of emergence is Jaegwon Kim’s argument that emergence is not possible because if only particles possess causal power, then the organization of particles cannot possess causal power beyond the particles themselves. According to contemporary physics, however, rather than particles, the fundamental constituents of the world are dynamic quantum fields, which are processes. Bickhard (2009) argues that in contrast to a substance/particle metaphysics, within a process metaphysics organization can have causal power and thus emergence is possible. Many sciences are moving toward a process perspective (Griffiths and Stotz, 2000; Bickhard, 2009), and that is what we are suggesting. We suggest that morality does not exist at the level of the individual, it emerges from the coordination of conflicting perspectives, which is possible within cooperative relationships based on mutual affection and respect. Thus, what emerges is a pattern of organization that exists at the level of the coordination of the views of the people involved (Boom, 2004; Bickhard, 2008, 2009, 2011).

2 We lack the space to critically examine all of the aspects of nativism that we see as problematic, nor can we examine all of the versions of nativism and their roots in the history of ideas (see Allen and Bickhard, 2013). But it is important to explicate some of the underlying assumptions of nativism and empiricism in an attempt to avoid the assimilation of our approach to the Procrustean bed of this worldview which contains only these two options. If we criticize nativism it might be assumed that we therefore must be endorsing empiricism. Instead, in taking an action-based approach we step off the pendulum between the two options and work from the perspective of a different worldview (Allen and Bickhard, 2013).

3 Graham et al. (2013) might respond that they were just using a metaphor, but they still need some actual biological process for getting from genes to neural interconnectivity that they claim is “*organized in advance of experience*” (p. 61, emphasis is original). They might respond that this is a problem for biologists not for them. However, the problem they give to biologists should at least be consistent with what is currently known about genetics and neural development (e.g., Stiles, 2009; Meaney, 2010; Stiles et al., 2015). Even if genes could “write … into neural tissue” by specifying neural interconnectivity it is not clear how many genes would be required to pre-specify the trillions of synaptic connections in human brains, and many species have more genes than humans. Infants are born with exuberant connectivity that is “pruned” away after birth in an ongoing dynamic and interactive process of brain development that does not seem consistent with Graham et al’s. (2013) claims (Mareschal et al., 2007; Stiles et al., 2015).

4 Our approach contrasts with the two steps in explaining moral obligation proposed by Tomasello (2020): first, the level of interpersonal obligation, and a second level of “objective” morality. He explains the second step as involving an extension to the collective resulting through social pressure experienced from the internalized “we.” In essence, this is conformity rather than morality. Although conformity is a part of human life, we suggest that this is an incomplete account of morality (Carpendale and Lewis, 2020).
